# Supplementary material for: Thermophilic Carboxylesterases from Hydrothermal Vents of the Volcanic Island of Ischia Active on Synthetic and Biobased Polymers and Mycotoxins
Source: Appl Environ Microbiol. 2023 Jan 31;89(2):e01704-22. doi: 10.1128/aem.01704-22 (PMC9972953; doi:10.1128/aem.01704-22)
Supplement: Supplemental file 1 — Supplemental material. Download aem.01704-22-s0001.pdf, PDF file, 2.9 MB [file aem.01704-22-s0001.pdf]

# Supporting information for the manuscript

## Thermophilic carboxylesterases from hydrothermal vents of the volcanic island of Ischia active on synthetic and biobased polymers and mycotoxins

Marco A. Distaso, Tatyana N. Chernikova, Rafael Bargiela, Cristina Coscolín, Peter Stogios, Jose L. Gonzalez-Alfonso, Sofia Lemak, Anna N. Khusnutdinova, Francisco J. Plou, Elena Evdokimova, Alexei Savchenko, Evgenii A. Lunev, Michail M. Yakimov, Olga V. Golyshina, Manuel Ferrer, Alexander F. Yakunin, and Peter N. Golyshin

## Supplementary Figures

**Figure S1.** The Shannon diversity index of the native and polyester enrichment cultures of the hydrothermal microbial communities of the volcanic island of Ischia.

**Figure S2.** Multiple sequence alignment of metagenomic carboxylesterases from Ischia with characterized homologous enzymes

**Figure S3.** Phylogenetic analysis of the metagenomic carboxylesterases from Ischia: **(A)**, IS10 and IS12; **(B)**, IS11

**Figure S4.** Carboxylesterase activity of IS10, IS11, and IS12: effect of pH, NaCl, and detergents.

**Figure S5.** Effect of organic solvents on the activity of IS10, IS11 and IS12.

**Figure S6.** Overall crystal structure of IS11: three views of the IS11 dimer related by 90º rotations.

**Figure S7.** Oligomeric state of purified Ischia carboxylesterases.

**Figure S8.** Structural models of IS10 (A) and IS12 (B) shown in three views related by a 90º rotation.

**Figure S9.** Structural models of IS10 and IS12: close-up view of the active sites.

## Supplementary Tables

**Table S1.** Environmental (*in situ*) and experimental conditions for metagenome samples and enrichment cultures.

**Table S2.** Esterase activities of purified Ischia carboxylesterases against various monoesters analyzed using a pH-shift assay.

**Table S3.** X-ray crystallographic statistics for the structure of IS11.

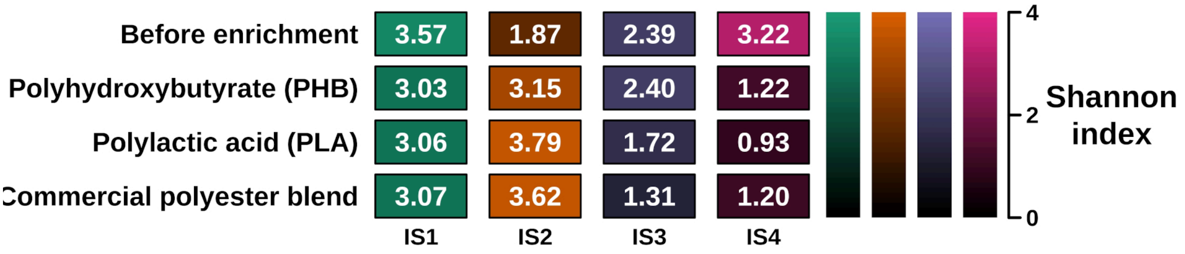


**Figure S1.** The Shannon diversity index of the native and polyester enrichment cultures of the hydrothermal microbial communities of the volcanic island of Ischia.

A

| | | | |
|---|---|---|---|
| | | S68 | |
| IS11 | 1 | MVTVNEVSDEARLRALVDRVTGLMTELGVPGVAVGILH GDREYTAGFGVTSLENPLPVTAGTLFQVGSIT | 70 |
| Pab87 | 1 | MD VGKLESFIVEKMAERKVPGISISIIKDGDVVYAKGFGYRNVEARLPSTPETIYGIGSIT | 61 |
| CcEstA | 1 | MTDITGVCPDRF AAVREVFEQNFADGGELGARFAFAIEGEVVVDLMGGFADRKRQVPFGPDTLTALFSTT | 70 |
| MGS0105 | 1 | MSNVQGYFDPRF ERVRELFAEQQQDEQARGAALCVTVGGDTVLDLWQGVTDKENQQVWEQDTLVNVFSCT | 70 |
| | | K71 | |
| IS11 | 71 | KTFTGTALMRLVEAGRIRLEDPVRKYLPDFRVKDASVSAAVTVRHLLTHTGGWVGDYFEDFGLG | 134 |
| Pab87 | 62 | KSFTALAIMKLVEEGGLSLDDPVEKFV NIKLRPFGEPVTVHHLLTHSSG IPSLGYA | 117 |
| CcEstA | 71 | KAVAALLIARLVDEGRLAYDQAVADVWPEF AQAGKDAVTVEQALSHQAGLS GFPDETDPAI | 131 |
| MGS0105 | 71 | KPLGAVALLQQVAAGRIELDAPLAEVWPEF AQAGKQSITLRQVLSHRSGLS AIAKALPPEA | 131 |
| | | Y160 N162 | |
| IS11 | 135 | DDALARMVAAMADLPQITP PDTVWAYNNAGF YVAGRIVEVVTGKPYESALR | 185 |
| Pab87 | 118 | EAFIDGMVGGDNWLPVSTPEETIAFARDMEKWAVAKPGERFFYLNTGY VLLGKIIEKVSGVSYEEYIK | 185 |
| CcEstA | 132 | WF DWDATCAKLAAMAPLFP IGSASGYHPVTYGYLAGEIFRRVDGRTMGTALR | 183 |
| MGS0105 | 132 | LF DWSTMSAALAEQAPWWE PGTAHGYAPVTYAWLLGEPLSRLTNESPGNYIQ | 183 |
| IS11 | 186 | ELVLDPLDLEHAYFFAHDV ISYRFAVGHLT GEAGP FVARPWALPRC | 231 |
| Pab87 | 186 | KKILEPLGMNRSYFFKEEVEKD KDVAMGYIL DKEGR LVPQPFPYGIT | 232 |
| CcEstA | 184 | EDICEPLGL DLWIGLPDS EHDRVADLM RPTAMPQFGEINP AVEA AFFKPWSSPGG | 238 |
| MGS0105 | 184 | QHICAPLGM DFHVGVPDK DLARIAHVS RLRNQSGDEGARKLFAAMGEPEGLTAKAFGNPVSMMTS | 248 |
| IS11 | 232 | ANPVGGLITSVRELFKYARFHLG SGPNILSAEARRLMQT PAGAADA | 277 |
| Pab87 | 233 | A DGGLLSSVLDLAKYLKMYIE RDESIVSKEYIEKMETSYIKVP W EIFG | 280 |
| CcEstA | 239 | KGAAEWRRVEIPSANGHATAPALARLMGALAHGGTLDGRSLITPAGIKAATAERLRGRDLVLP Y | 302 |
| MGS0105 | 249 | TNKREWQQAEILSANGTGNARSLARFWQLLAHGGELDRVKLLDSELVSLMQQEHSQGQDRTLL C | 312 |
| | | H299 | |
| IS11 | 278 | GRLMGITWFIQ PVDGDIRLVGHGGGTNGQITLFNFVPGRNFAVCVLTNSDRGGAINAEVTRWAVE | 342 |
| Pab87 | 281 | GEGYGYGLIIY PNFLGEKLVGHSGSVGMYTGYIGYIPEKKIGVAVLENSSGYPP SYIAMYALA | 343 |
| CcEstA | 303 | EISWGAGFMRNE PNFYYGPTAEAFGHSGWGG SCAFADPTRGVSGAYVMNKQGNALIGDPRSVRLIE | 368 |
| MGS0105 | 313 | PTRFGLGVMLEQDAPGGGFGMGPQAFGHPGAGG SLGFCDPEAKVGFGYVTNTMGPYVLMDPRALALSQ | 380 |
| IS11 | 343 | SFLGAA WPEPEIYSLPPER LATWAGRYEGVLSDI ELVLEGDWLVLKQTPKGGFPRKDSPPRPAPPP | 408 |
| Pab87 | 344 | LLLGKN PEKELPFIYRERILKKVEGRYMGYKGTIKFEVKVDGDVVYLRALGRAF | 397 |
| CcEstA | 369 | AAYASL | 374 |
| MGS0105 | 381 | AVHDCLRELD | 390 |
| IS11 | 409 | SRLGFYAPDRVVALD LPLK GSRGE FLRGPDGSIAWFRFGGRLFRPGQ | 455 |
| Pab87 | 398 | TYTIPLFPEVLEEDFIKCYTLSNGRKMYAEFYIK DNKVDLIFERYRLIKS | 447 |
| CcEstA | 375 | | |
| MGS0105 | 391 | | |

B

| | | | |
|---|---|---|---|
| IS10 | 1 | MALYPSARWVLDRMAERGTRPFNEVPVERARQQFDA MMAGAPR PEVARTEDRTVPGPAGDIPVRIYWPQQ QTLPL | 75 |
| IS12 | 1 | MALDPGAAALLQQMAQAGVPPFNQMTVQQARQSMTG LAGTAPK YPVAKVEDRTIPGPAGQIPVRIYWPIS GQKVG | 75 |
| Est8 | 1 | MALDPQAKGLLDAMAANPAPRIIDLPVKEAREMYRG IAAQLDLQDLPIGKTEDRKIPGPAGDIPVRIYTPVAAGGAALP | 79 |
| EstE1 | 1 | MPLDPQIKPILERIRALSI AASPQELRRQVEEQSRLLTAAVQ EPIAETRDVHIPVSGGSIRARVYFPKK AAGLP | 74 |
| PestE1 | 1 | MPLSPILRQILQQLAAQ LQFRPDMDVKTVREQFEKSSLILVKMAN EPIHRVEDITIPGRGGPIRARVYRPRD GERLP | 77 |
| | | S155 | |
| IS10 | 76 | LLVYFHGGGWIMGSIESHDPTCRAIANVAGCIVVSVGYRLAPEHKFPEPLEDCYAAVRWAEENAASLGADPSLLAVGGDSAG | 157 |
| IS12 | 76 | LVVYYHGGGWVICDLETHDGTCRQLANASDCIVMSVDYRLAPEHKFPAAVEDCYAAAAWAAENAATFGVDPSRLVVAGDSAG | 157 |
| Est8 | 80 | VLVYFHGGGWVIGDLETHDALCRSFANEAGCKVVAVDYRLAPEHRFPAAAEDCLAAVKWVETNASEIGVDANRIAVAGDSAG | 161 |
| EstE1 | 75 | AVLYYHGGGFVFGSIETHDHICRRLSRLSDSVVVSVDYRLAPEYKFPTAVEDAYAALKWVADRADELGVDPDRIAVAGDSAG | 156 |
| PestE1 | 78 | AVVYYHGGGFVLGSVETHDHVCRRLANLSGAVVVSVDYRLAPEHKFPAAVEDAYDAAKWVADNYDKLGVDNGKIAVAGDSAG | 159 |
| IS10 | 158 | GNLAAAVSLLARDRGGPRLAHQVLAYPVLSHGAA FPSYEENA DGYFLTREMMLYCWRQYLRDEKDGDDPYASPLKASEL | 236 |
| IS12 | 158 | GNLAAVVALLAKERGGPRLAHQVLIYPVTDYSFD RPSYRENA EGYLLTRDAMEWFWRHYLNSDADGQNPLASPLRAPDL | 236 |
| Est8 | 162 | GNLAAVVSQLALAAKGPRIAFQLLIYPVTDTNVD TASYRENA SGYFLERDGMIWFFDHYLNG ADRTDPRVAPLRAASL | 239 |
| EstE1 | 157 | GNLAAVVSILDRNSGEKLVKKQVLIYPVVNMTGVPTASLVEFGVAETTSLPIELMVWFGRQYLKRPEEAYDFKASPLLA DL | 237 |
| PestE1 | 160 | GNLAAVTAIMARDRGESFVKYQVLIYPAVNLTGSPTVSRVEYSGPEYVILTADLMAWFGRQYFSKPQDALSPYASPIFA DL | 240 |
| | | D250 H280 | |
| IS10 | 237 | SGLPTATIITAEHDPLRDEAEAYGVRLAQAGVPNYCLRYVGMIHGFLSLDNLLPPGRHALWSVATGLRLAATETH KRA | 314 |
| IS12 | 237 | RGLPSATVITAEYDPLRDEGEAYAQRLMEAGVPTTCVRYLGQIHGFVTLEHVLPAGRQALLALGATIKGVLTGALGGQPAAA | 318 |
| Est8 | 240 | AGLPRAYVITAGFDPLKDEGRAYAEALKAAGVPTEYVNYEGMIHGFFNLQAAFDVSRDAVKAAAKALKEALA | 311 |
| EstE1 | 238 | GGLPPALVVTAEYDPLRDEGELYAYKMKASGSRAVAVRFAGMVHGFVSFYPFVDAGREALDLAAASIRSGLQPS | 311 |
| PestE1 | 241 | SNLPPALVITAEYDPLRDEGELYAHLLKTRGVRAVAVRYNGVIHGFVNFYPILEEGREAVSQIAASIKSMAVA | 313 |

**Figure S2.** Multiple sequence alignment of metagenomic carboxylesterases from Ischia with characterized homologous enzymes. (A), IS11; (B), IS10 and IS12. The following proteins are shown: (A), IS11, Pab87 from *Pyrococcus abyssi* (Q9V2D6), MGS0105 from a cold marine metagenome (T1W153), and CcEstA from *Caulobacter crescentus* (Q9ABH2). The IS11 catalytic residues are coloured red and labelled, whereas conserved residues are shown in bold. The IS11 secondary structure elements are shown above the alignment. (B), IS10, IS12, Est8 from an oil-degrading metagenome (ALO81572), EstE1 from a thermophilic metagenome (Q5G935), and PestE1 from *Pyrobaculum calidifontis* (Q8NKS0). The catalytic residues of IS10 are coloured red and labelled, whereas the conserved residues are shown in bold. The secondary structure elements of IS11 (A) and IS10 (B) are shown above the alignments. Both sequence alignments were prepared using the MAFFT online tool (https://mafft.cbrc.jp/alignment/server/ ) and STRAP (http://www.bioinformatics.org/strap/ ).

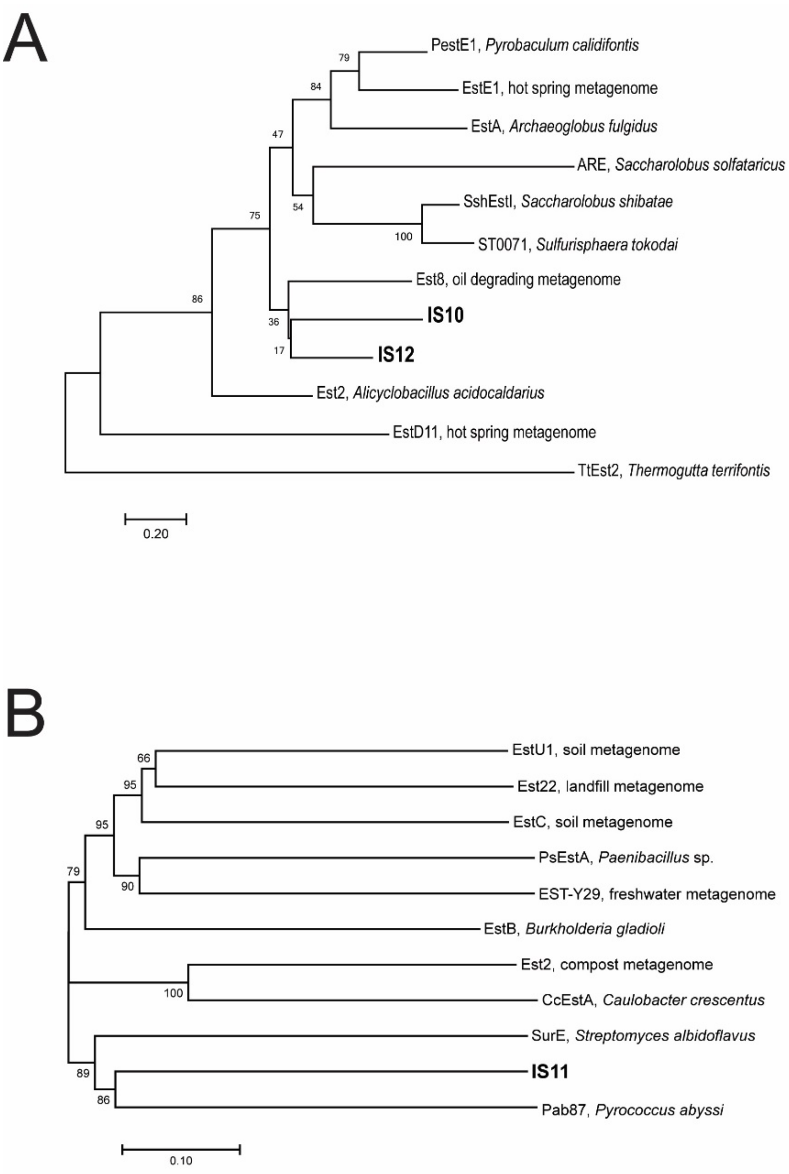


**Figure S3.** Phylogenetic analysis of the metagenomic carboxylesterases from Ischia: **(A)**, IS10 and IS12; **(B)**, IS11. The phylogenetic tree showing the relationship between IS10, IS11, IS12, and biochemically characterized carboxylesterases from various metagenomes and microorganisms. The tree was generated with a MEGA7 software using the Neighbour-Joining method (see Materials and Methods for details). The bootstraps values (for 1,000 pseudoreplicates) are indicated at the branches, and scale bars indicate 0.2 and 0.1 substitutions per position in (A) and (B), respectively. The following proteins were used for the analysis: (A), PestE1 from *Pyrobaculum calidifontis* (Q8NKS0), EstE1 from a hot spring metagenome (Q5G935), EstA from *Archaeoglobus fulgidus* (O28558), ARE from *Saccharolobus solfataricus* (B5BLW5), SshEstI from *Saccharolobus shibatae* (Q5NU42), ST0071 from *Sulfurisphaera tokodai* (Q976W8), Est8 from an oil degrading metagenome (ALO81572), Est2 from *Alicyclobacillus acidocaldarius* (Q7SIG1), EstD11 from a hot spring metagenome (A0A8I3AZT4), TtEst2 from *Thermogutta terrifontis* (KT724966); (B), EstU1 from a soil metagenome (AFU54388), Est22 from a landfill metagenome (KF052088), EstC from a soil metagenome (ACH88047), PsEstA from *Paenibacillus* sp. (AHL66978), EST-Y29 from a freshwater metagenome (NBR33940), EstB from *Burkholderia gladioli* (Q9KX40), Est2 from a compost metagenome (ALH24853), CcEstA from *Caulobacter crescentus* (Q9ABH2), SurE from *Streptomyces albidoflavus* (A0A679G4U8), Pab87 from *Pyrococcus abyssi* (Q9V2D6).

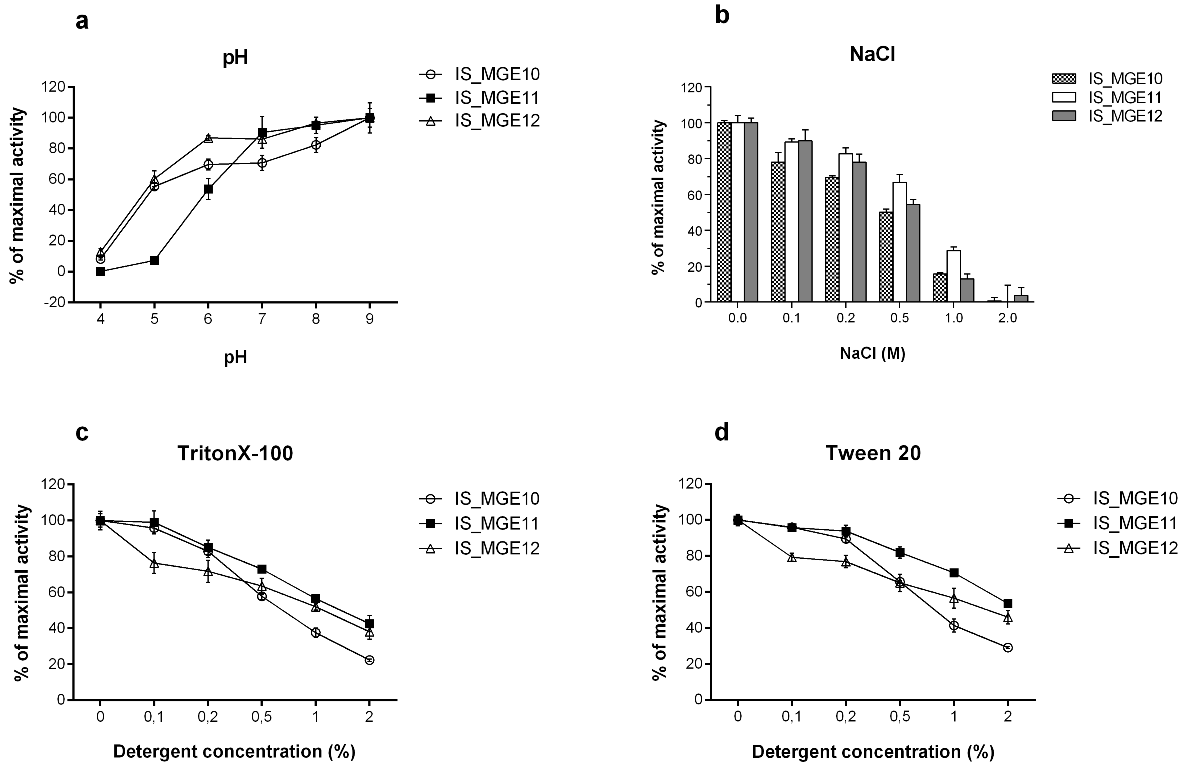


**Figure S4.** Carboxylesterase activity of IS10, IS11, and IS12: effect of pH, NaCl, and detergents. Esterase activity of purified proteins was measured as indicated using *p*NP-butyrate as substrate at 30ºC as described in Materials and Methods. Results are means ± SD from three independent experiments.

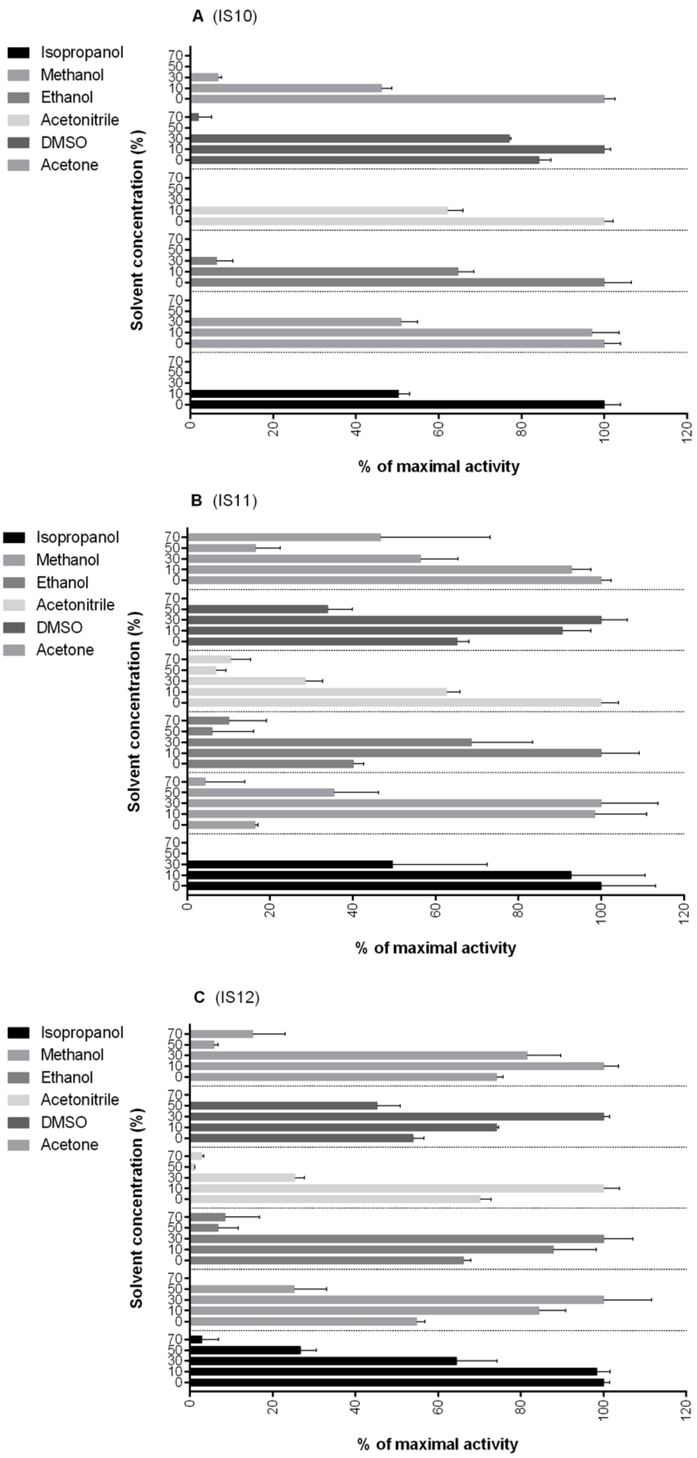


**Figure S5.** Effect of organic solvents on the activity of IS10, IS11 and IS12. Carboxylesterase activity of purified proteins was measured in the presence of increasing solvent concentrations (10, 30, 50, 70 %, v/v) using *p*NP-butyrate as substrate at 30 ºC. 100 % activity correspond to enzyme activity assayed in 50 mM potassium phosphate buffer (pH 7.0) at 30 ºC, as described in Materials and Methods. Results are means ± SD from three independent experiments.

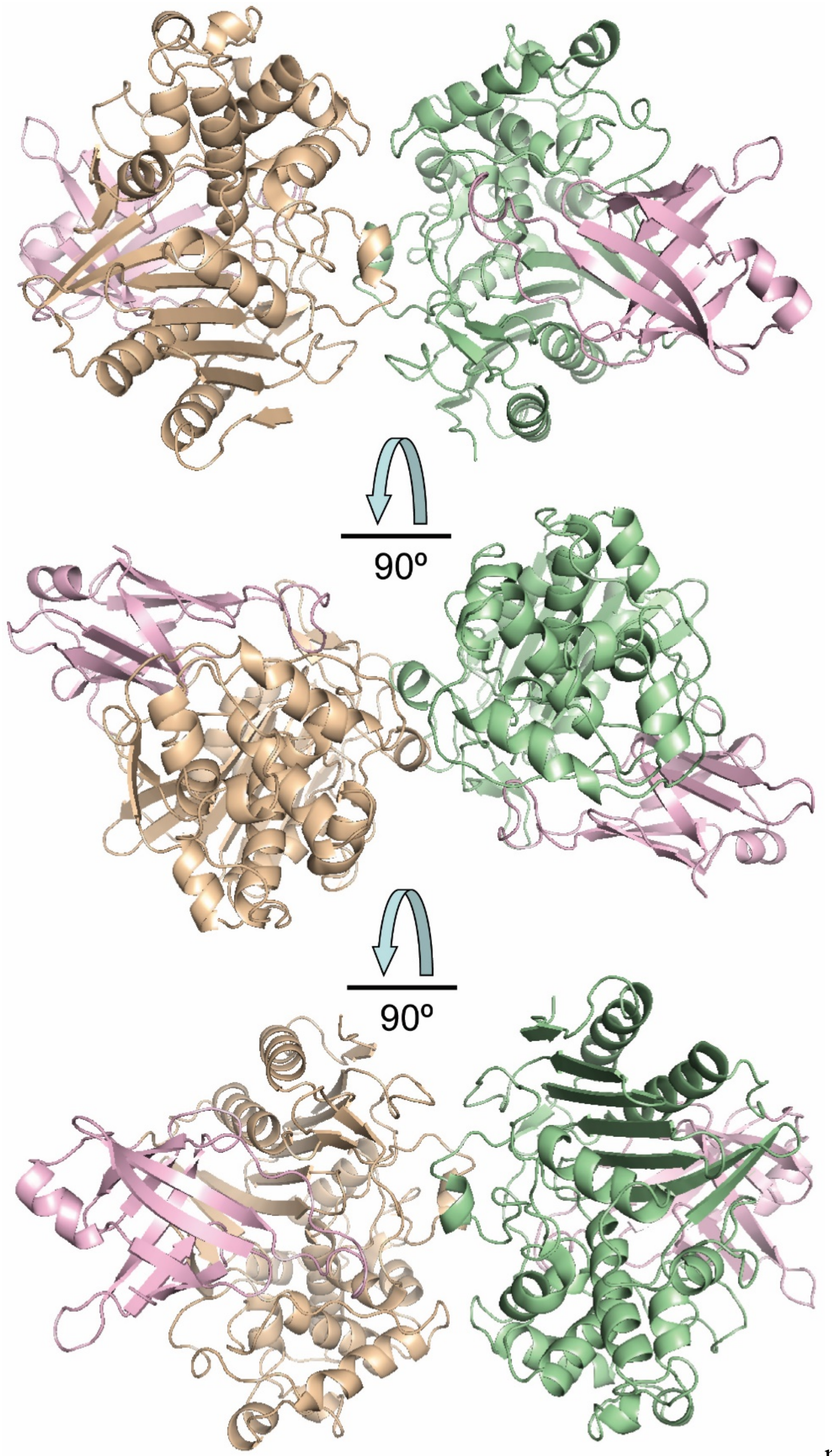


m

**Figure S6.** Overall crystal structure of IS11: three views of the IS11 dimer related by 90º rotations. The protein subunits are shown as ribbon diagrams with the core domains coloured wheat or green, whereas their lipocalin domains are coloured pale pink.

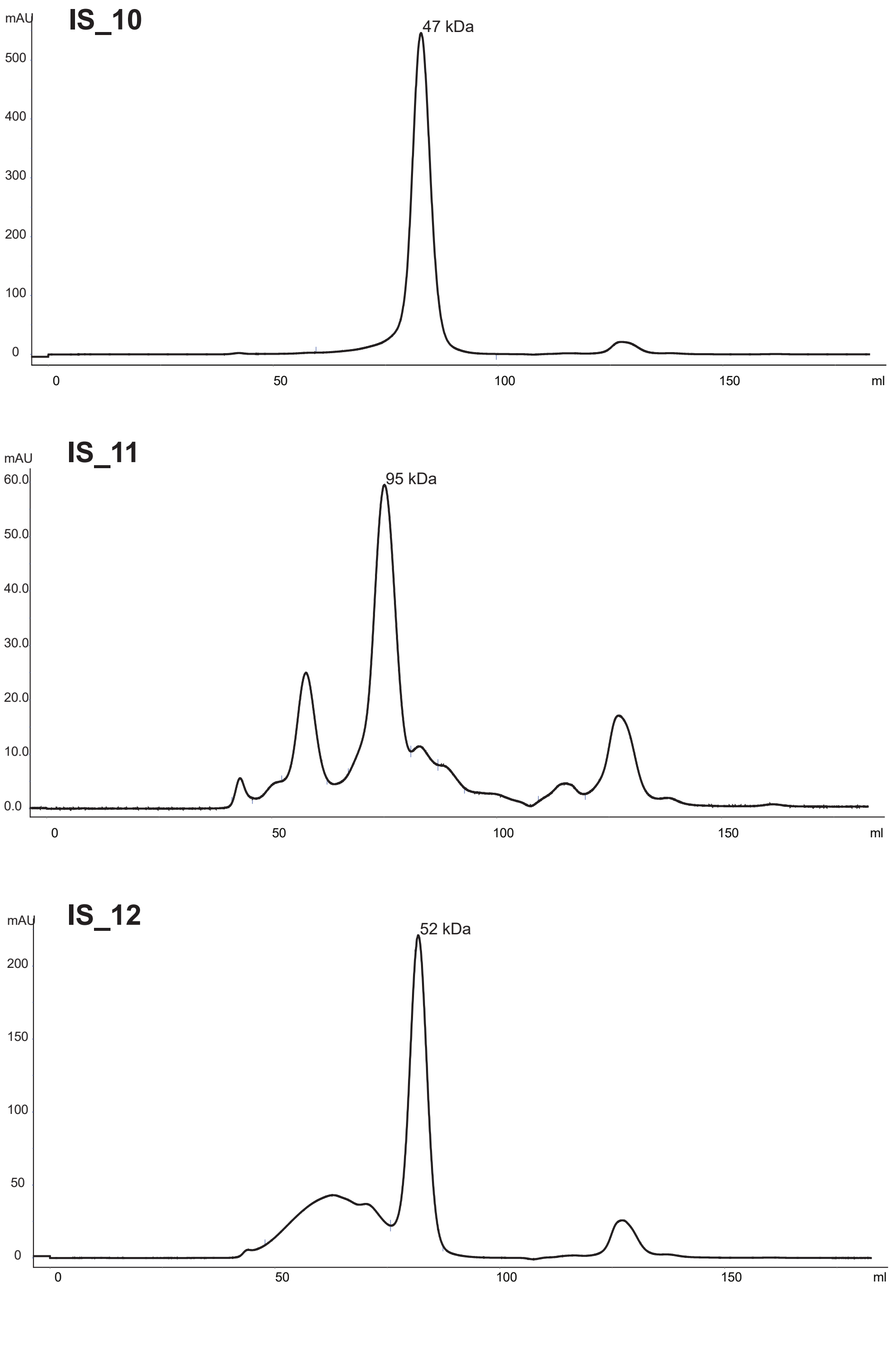


**Figure S7.** Oligomeric state of purified Ischia carboxylesterases. Elution profiles for purified proteins from size-exclusion chromatography using a Superdex 200 10/300 GL column. The numbers on the protein peaks indicate elution volumes (in ml), and corresponding molecular mass is reported (predicted monomer mass: 34.3 kDa for IS10, 49.4 kDa for IS11, and 33.9 kDa for IS12).

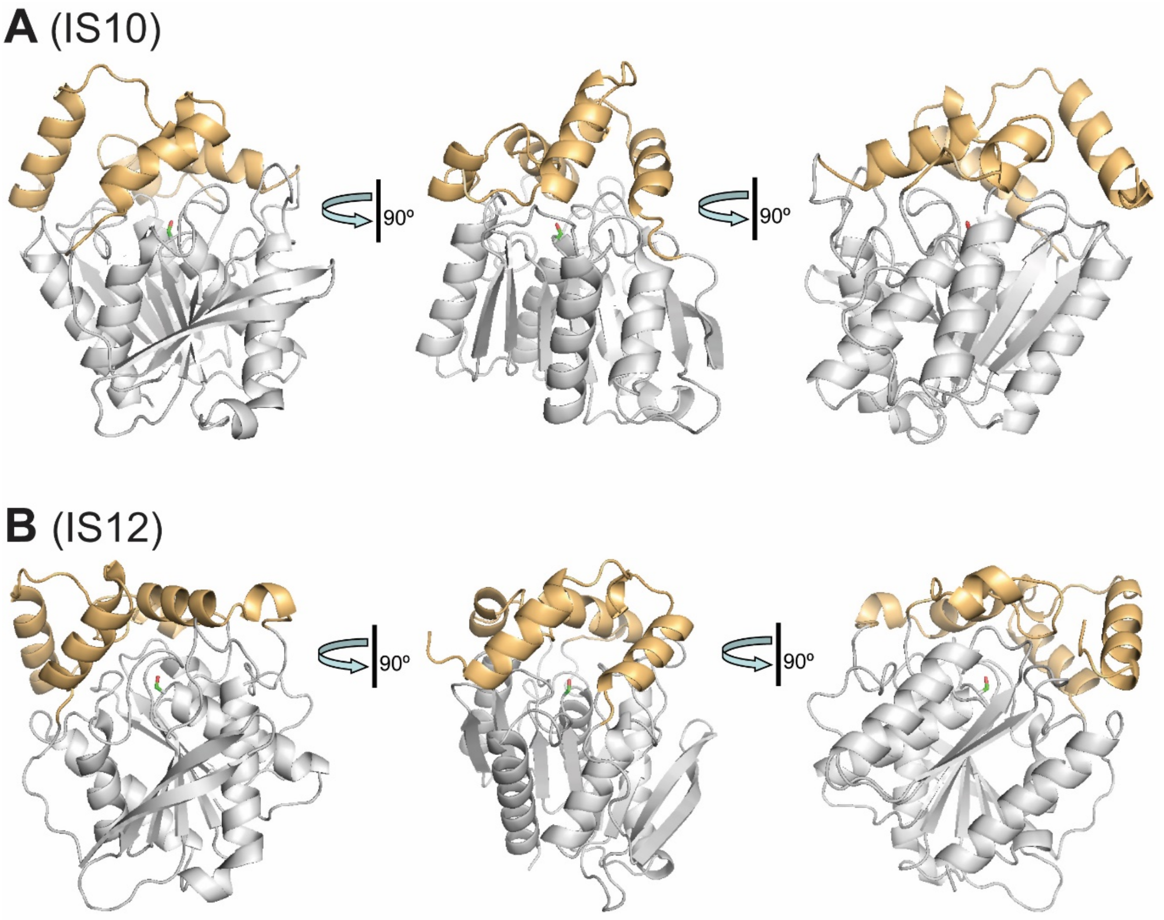


**Figure S8.** Structural models of IS10 (A) and IS12 (B) shown in three views related by a 90º rotation. Structural models were generated using the Phyre2 protein fold recognition server (1). Both models were constructed using the crystal structure of metagenomic carboxyl esterase Est8 (PDB code 4YPV (2) as the template (51% and 55% of sequence identity to IS10 and IS12, respectively; 100% confidence). The protein lid domain is coloured in light orange, whereas the core domain is shown in grey with the position of the active site indicated by the side chain of catalytic Ser155.

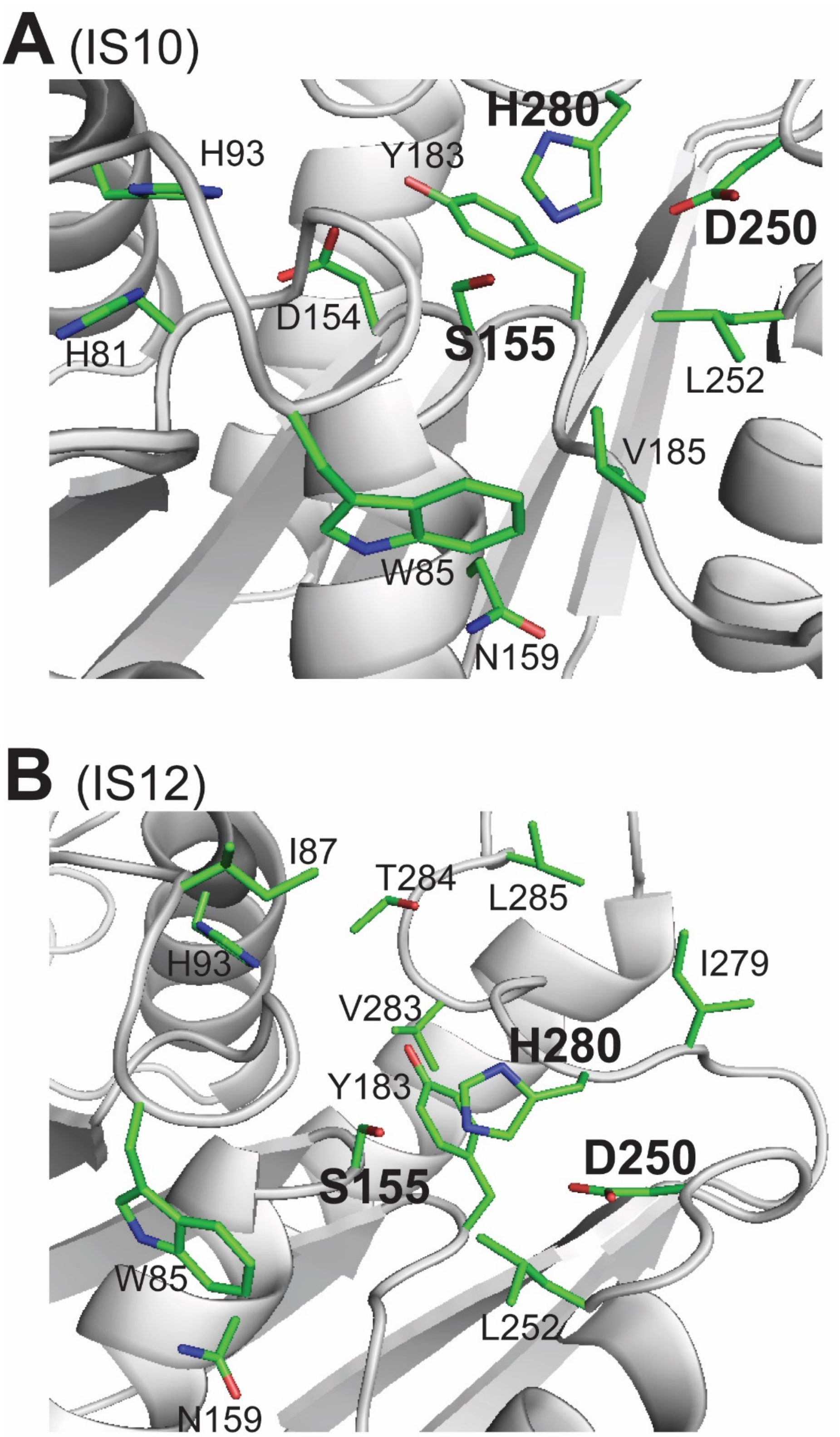


**Figure S9.** Structural models of IS10 and IS12: close-up view of the active sites. The protein ribbon is coloured in grey with amino acid side chains shown as sticks and carbon atoms coloured in green. The catalytic triad residues are indicated using larger labels (Ser155, His280, and Asp250), whereas other residues are potentially involved in substrate binding.

**Table S1.** Environmental (*in situ*) and experimental conditions for metagenome samples and enrichment cultures.

| Sampling Site | | | | Enrichment Culture | | | |
|---|---|---|---|---|---|---|---|
| **Name** | **ID** | **Temp** | **pH** | **Medium** | **pH** | **Substrate** | **Temp** |
| Cavascura | IS1 | 45°C | 8.5 | DSMZ 1374 | 7.5 | 1. PHB<br>2. PLA<br>3. Blend | 50°C |
| | IS2 | 55°C | 7.0 | DSMZ 1374 | 7.5 | 1. PHB<br>2. PLA<br>3. Blend | 50°C |
| Maronti Beach | IS3 | 75°C | 4.5 | DSMZ 88 | 4.5 | 1. PHB<br>2. PLA<br>3. Blend | 75°C |
| | IS4 | 85°C | 5.0 | DSMZ 88 | 4.5 | 1. PHB<br>2. PLA<br>3. Blend | 75°C |

[a] Polyester substrates used in enrichment cultures: PHB, polyhydroxybutyrate; PLA, polylactic acid; Blend, commercial polyester blend.

**Table S2.** Esterase activities of purified Ischia carboxylesterases against various monoesters analysed using a pH-shift assay (see Materials and Methods for details).

| **Substrates** | **Activity**, U/mg (± SD) | | |
|---|---|---|---|
| | **IS10** | **IS11** | **IS12** |
| 1. (1*R*)-(-)-Menthyl acetate | 23.2 ± 0.7 | n.d. | 29.6 ± 0.9 |
| 2. (1*S*)-(+)-Menthyl acetate | 28.3 ± 0.8 | n.d. | 31.3 ± 0.9 |
| 3. 1-Naphthyl acetate | 2,576 ± 64 | 14.2 ± 1.9 | 1,414 ± 0.4 |
| 4. 1-Naphthyl butyrate | 353 ± 10.3 | 28.3 ± 1.4 | 506 ± 0.9 |
| 5. (1*R*)-(+)-Neomenthyl acetate | 20.6 ± 0.6 | n.d. | 27.0 ± 0.8 |
| 6. (1*S*)-(+)-Neomenthyl acetate | 11.6 ± 0.3 | n.d. | 14.2 ± 0.4 |
| 7. 2,4-Dichlorobenzyl 2,4-dichlorobenzoate | 151 ± 4.5 | n.d. | 38.6 ± 1.2 |
| 8. 3-Methyl-3-buten-1-yl acetate | 10.3 ± 0.3 | 2.6 ± 0.1 | 103 ± 3.0 |
| 9. Benzyl (*R*)-(+)-2-hydroxy-3-phenylpropionate | 28.3 ± 0.8 | 398 ± 8.9[b] | 29.6 ± 11.9 |
| 10. Butyl acetate | 136± 0.5 | n.d. | 145 ± 0.5 |
| 11. Cyclohexyl butyrate | 403 ± 12 | n.d. | 380 ± 11.4 |
| 12. Ethyl acetoacetate | 15.4 ± 0.5 | 1.3 ± 0.1 | 16.7 ± 0.5 |
| 13. Ethyl 2-chlorobenzoate | 131 ± 4.0 | n.d. | 149 ± 4.6 |
| 14. Ethyl (*R*)-(+)-4-chloro-3-hydroxybutyrate | 3.9 ± 0.2 | 24.4 ± 0.3 | 7.7 ± 0.8 |
| 15. Ethyl (*S*)-(−)-4-chloro-3-hydroxybutyrate | 19.3 ± 0.7 | 14.2 ± 0.7 | 23.8 ± 0.5 |
| 16. Ethyl 2-ethylacetoacetate | 16.7 ± 0.5 | 20.6 ± 0.6 | 20.6 ± 0.6 |
| 17. (+)-Ethyl D-lactate | 18.0 ± 0.6 | 28.9 ± 0.9 | 28.3 ± 0.9 |
| 18. (−)-Ethyl L-lactate | 25.7 ± 0.7 | 6.4 ± 0.8 | 25.7 ± 0.2 |
| 19. Ethyl 2-methylacetoacetate | 27.0 ± 0.8 | 10.3 ± 0.3 | 29.7 ± 0.8 |
| 20. Ethyl 3-oxohexanoate | 25.7 ± 0.8 | 9.0 ± 0.3 | 27.0 ± 0.8 |
| 21. Ethyl propionylacetate | 26.1 ± 0.8 | 18.0 ± 0.5 | 28.3 ± 0.8 |
| 22. Geranyl acetate | 467 ± 12.6 | 27.0 ± 0.9 | 520 ± 14.4 |
| 23. Glucose pentaacetate | 284 ± 8.8 | n.d. | 328 ± 10.4 |
| 24. Glyceryl triacetate | 979 ± 27.6 | 9.0 ± 0.3 | 1,072 ± 3.0 |
| 25. Glyceryl tributyrate (tributyrin) | 1,100 ± 32.0 | 30.9 ± 1.0 | 1,558 ± 47.9 |
| 26. Glyceryl tripropionate | 3,430 ± 88.9 | 32.2 ± 5.4 | 2,950 ± 1.0 |
| 27. Hexyl acetate | 34.7 ± 1.0 | n.d.[a] | 1.3 ± 0.1 |
| 28. Methyl glycolate | 3.9 ± 0.1 | n.d. | 6.4 ± 0.2 |
| 29. (−)-Methyl (*R*)-3-hydroxyvalerate | 12.9 ± 0.4 | 6.4 ± 0.5 | 15.4 ± 0.3 |
| 30. (+)-Methyl (*S*)-3-hydroxyvalerate | 6.4 ± 0.2 | 20.6 ± 0.3 | 7.7 ± 0.6 |
| 31. Octyl acetate | 154 ± 4.4 | n.d. | 129 ± 3.8 |
| 32. n-Pentyl benzoate | 16.7 ± 0.5 | n.d. | 14.4 ± 0.4 |
| 33. Phenyl acetate | 10,456 ± 15.7 | 21.9 ± 0.6 | 3,375 ± 15.4 |

| | | | |
|---|---|---|---|
| 34. Phenyl propionate | 8,792 ± 41.7 | 25.7 ± 0.8 | 8,219 ± 43.0 |
| 35. Phthalic acid diethyl ester | 29.6 ± 0.9 | n.d. | 106 ± 3.2 |
| 36. Propyl acetate | 20.6 ± 0.7 | n.d. | 25.7 ± 0.6 |
| 37. Propyl butyrate | 34.7 ± 0.1 | n.d. | 50.2 ± 1.5 |
| 38. Propyl hexanoate | 246 ± 7.0 | n.d. | 305 ± 8.8 |
| 39. γ-Valerolactone | 172 ± 5.4 | 60.5 ± 2.4 | 219 ± 6.7 |
| 40. Vinyl acetate | 74.6 ± 0.2 | n.d. | 66.9 ± 0.1 |
| 41. Vinyl benzoate | 1,100 ± 32.8 | n.d. | 1,085 ± 32.0 |
| 42. Vinyl butyrate | 201 ± 5.7 | n.d. | 616 ± 18.3 |
| 43. Vinyl crotonate | 5.1 ± 0.2 | n.d. | 90.1 ± 2.7 |
| 44. Vinyl propionate | 188 ± 5.5 | n.d. | 834 ± 24.0 |

[a] n.d., not detectable.
[b] The hydrolysis of benzyl (R)-(+)-2-hydroxy-3-phenylpropionate yields (*R*)-(+)-2-Hydroxy-3-phenylpropionic acid, which is an antimicrobial compound with broad spectrum activity (3).

**Table S3.** X-ray crystallographic statistics for the structure of IS11.

| **PDB code** | **7SPN** |
|---|---|
| **Data collection** | |
| Space group | P2 |
| Unit cell | |
| *a, b, c* (Å) | 50.22, 103.36, 94.86 |
| α, β, γ, (°) | 90, 126.56, 90 |
| Resolution, Å | 50.00 – 2.92 |
| $R_{merge}$[a] | 0.115 (0.831)* |
| $R_{pim}$[b] | 0.069 (0.528) |
| $CC_{1/2}$ | 0.993 (0.572) |
| $I / \sigma(I)$ | 11.96 (1.10) |
| Completeness, % | 98.4 (97.6) |
| Redundancy | 3.6 (3.2) |
| | |
| **Refinement** | |
| Resolution, Å | 45.09 – 2.92 |
| No. unique reflections: working, test | 18739, 1834 |
| *R*-factor/free *R*-factor[c] | 20.7/26.0 (30.8/38.2) |
| No. refined atoms, molecules | |
| Protein | 6990, 2 |
| Water | 85 |
| *B*-factors | |
| Protein | 71.3 |
| Water | 64.0 |
| r.m.s.d. | |
| Bond lengths, Å | 0.002 |
| Bond angles, ° | 0.534 |

*values in brackets refer to highest resolution shells.

[a]$R_{merge} = \Sigma_{hkl}\Sigma_j|I_{hkl.j} - \langle I_{hkl}\rangle|/\Sigma_{hkl}\Sigma_j I_{hk,j}$, where $I_{hkl,j}$ and $\langle I_{hkl}\rangle$ are the *j*th and mean measurement of the intensity of reflection *j*.

[b]$R_{pim} = \Sigma_{hkl}\sqrt{(n/n-1)}\ \Sigma^{n}_{j=1}|I_{hkl.j} - \langle I_{hkl}\rangle|/\Sigma_{hkl}\Sigma_j I_{hk,j}$

[c]$R = \Sigma|F_p^{obs} - F_p^{calc}|/\Sigma F_p^{obs}$, where $F_p^{obs}$ and $F_p^{calc}$ are the observed and calculated structure factor amplitudes, respectively.

## Supplementary References


1. Kelley LA, Mezulis S, Yates CM, Wass MN, Sternberg MJ. 2015. The Phyre2 web portal for protein modeling, prediction and analysis. *Nat Protoc* **10,** 845-858.
2. Pereira MR, Maester TC, Mercaldi GF, de Macedo Lemos EG, Hyvönen M, Balan A. 2017. From a metagenomic source to a high-resolution structure of a novel alkaline esterase. *Appl Microbiol Biotechnol* **101**, 4935-4949.
3. Zhu YB, Xu Y, Wang LM, Qi B. 2018. Biosynthesis of (R)-2-hydroxy-3-phenylpropionic acid using whole recombinant Escherichia coli cells in an aqueous/n-octane biphasic system. *J Zhejiang Univ Sci B* **19**, 285-292.
